# Mitochondrial dysfunction is a hallmark of woody breast myopathy in broiler chickens

**DOI:** 10.3389/fphys.2025.1543788

**Published:** 2025-02-17

**Authors:** Elizabeth S. Greene, Paula R. Chen, Carrie Walk, Mike Bedford, Sami Dridi

**Affiliations:** ^1^ Center of Excellence for Poultry Science, Division of Agriculture, University of Arkansas, Fayetteville, AR, United States; ^2^ USDA-ARS, Plant Genetics Research Unit, Columbia, MO, United States; ^3^ AB Vista, Marlborough, United Kingdom

**Keywords:** woody breast, broiler, mitochondrial dysfunction, hypoxia, bioenergetics

## Abstract

The woody breast (WB) myopathy poses significant economic and welfare concerns to the poultry industry, however, there is no effective strategy to mitigate this pathology due to its unknown etiology. After showing previously that hypoxia is a key factor in WB progression, we used here various techniques demonstrating dysregulated mitochondria (morphology, biogenesis, tethering, function, and bioenergetics) in WB-affected muscles and in hypoxic myoblasts compared to healthy tissues and normoxic cells, respectively. The increased levels of calcium (Ca^2+^) in both WB-affected tissues and hypoxic myoblasts suggested that mitochondrial Ca^2+^ overload is likely a leading cause for mitochondrial dysfunction that merits further in-depth investigation. These findings are the first, to the best of our knowledge, to provide fundamental insights into the underlying molecular mechanisms of WB and open new vistas for understanding the interplay between calcium, mitochondrial (dys)function, and avian muscle health for subsequent development of effective preventative/corrective strategies.

## 1 Introduction

Concurrent with an increasing global population, demand for poultry, and specifically chicken meat, is projected to increase over the coming decades (Dohlman et al., 2024). Though selection for increased growth, efficiency, and meat yields has made incredible progress to date, this trajectory must be maintained and improved. One challenge, however, has been the parallel rise in growth-related abnormalities that impact production, welfare, and sustainability. The woody breast (WB) myopathy in chicken breast meat was initially described over 10 years ago (Sihvo et al., 2014), and its occurrence is surging at global scale, already present in many world regions (de Brot et al., 2016; Cemin et al., 2018; Kawasaki et al., 2018). It is deleteriously impacting global chicken meat production and quality, leading to downgraded value, meat condemnation, and increased processing charges, all together results in heavy economic losses (Soglia et al., 2016).

A plethora of research since its initial identification has sought to define causes and potential preventive-corrective measures. Characterized by a noticeable hardness of the *Pectoralis major* muscle (Sihvo et al., 2014), with muscle fiber degeneration, necrosis, lipidosis, and fibrosis evident upon histological examination (Sihvo et al., 2014; Velleman and Clark, 2015), WB presents also a significant wellbeing concern to the poultry industry. Although the underlying mechanisms of this myopathy yet to be fully characterized, work by our group (Greene et al., 2019; Emami et al., 2021) and others (Zhang et al., 2024) indicates a hypoxic state in WB muscles, associated with increased muscle fiber size, decreased capillary density, and therefore, decreased oxygen supply and clearance of metabolic waste products from the tissue. Additionally, in skeletal muscle, it has been established that chronic hypoxic conditions lead to the production of mitochondrial reactive oxygen species (ROS) and subsequent oxidative stress (Clanton, 2007), all factors likely contributing to the WB myopathy.

A major function of mitochondria is the generation of adenosine triphosphate (ATP) for energy through oxidative phosphorylation (OXPHOS). The mitochondria of skeletal muscle are also a primary source of ROS, as well as the major target of oxidative damage and the intracellular redox buffering system (Murphy, 2009), and mitochondrial dysfunction has been identified as an underlying factor in multiple muscular diseases (Chen et al., 2022). This dysfunction can be caused by defective OXPHOS (Fernandez-Vizarra and Zeviani, 2021), mitochondrial DNA (mtDNA) mutations (Rossignol et al., 2003), Ca^2+^ imbalances (Garbincius and Elrod, 2022), and structural defects (Vincent et al., 2016; Jenkins et al., 2024). In addition, an imbalance between mitochondrial fusion and fission (Sprenger and Langer, 2019), lysosomal dysfunction (de la Mata et al., 2016), and defects in mitophagy (Gottlieb et al., 2011) can lead to mitochondrial damage. However, most of this in-depth relationship between mitochondrial (dys)function and muscle health has been elucidated in human myopathies and murine models. We hypothesized that mitochondrial dysfunction is also a key contributor to the WB condition, which is a distinct myopathy peculiar and unique to poultry. By using *in vivo*-derived samples and a highly relevant *in vitro* primary cell culture model (Greene et al., 2023), we showed a disproportional mitochondrial morphology along with dysregulated function, bioenergetics, and dynamics in WB-affected muscle and hypoxic primary myotubes.

## 2 Materials and methods

### 2.1 Care and use of animals

This study was conducted in accordance with the National Institutes of Health recommendations guide for laboratory animal use and care. All the procedures in this study were approved by the University of Arkansas Animal Care and Use Committee under protocol #21050. Day-old male Cobb 500 broiler chicks (n = 720) were reared in floor pens covered with clean pine wood shavings and equipped with separate feeders and water lines in a controlled environment. Ambient temperature was gradually reduced from 32°C to 25°C by day 21. A 23 h light/1 h dark cycle and a ∼30–40% relative humidity was maintained throughout the experiment. Birds were fed a nutrient adequate diet, recommended by the poultry industry and formulated to meet Cobb 500 nutrition requirements, with starter, grower, and finisher phases.

### 2.2 Processing and WB myopathy scoring

Birds (n = 512) were processed on day 56 at the University of Arkansas Pilot Processing Plant (Fayetteville, AR) using a commercial inline system. Feed was removed 10 h prior to processing, while *ad libitum* access to water was maintained. Birds were electrically stunned, exsanguinated, soft scalded, de-feathered, and eviscerated, then chilled for 4 h prior to deboning. Breast fillets were hand scored by a well-trained person for WB on a scale of 0–3, with 0 showing no signs of WB, 1 was mild, 2 was considered moderate, and score 3 being severe WB (Kuttappan et al., 2016; Dalgaard et al., 2018; Greene et al., 2019). Breast muscle samples taken from the cranial region of score 0 (normal) and 3 (severe WB) were either snap-frozen in liquid nitrogen and stored at −80 °C for RNA and protein analysis or fixed for electron microscopy.

### 2.3 Chicken primary myoblast culture

Chicken primary myoblasts were isolated from E18 embryos as previously described (Greene et al., 2023). Cells were cultured at 37°C in a humidified atmosphere in complete media for the indicated times. Hypoxia was induced by placing the cultures into a gas-tight hypoxic chamber (1% O_2_/5% CO_2_/94% N_2_; The Baker Company, Inc., Sanford, ME) for 24 h. The control cells were maintained at normoxic conditions (5% CO_2_/95% O_2_).

### 2.4 Transmission electron microscopy

Unless otherwise stated, all reagents were purchased from Electron Microscopy Sciences and all specimen preparation was performed at the Electron Microscopy Core Facility, University of Missouri. Samples were fixed in 2% paraformaldehyde, 2% glutaraldehyde in 100 mM sodium cacodylate buffer, pH 7.35. Tissues were rinsed with 100 mM sodium cacodylate buffer, pH 7.35 (Sigma Aldrich, St. Louis, MO) and 130 mM sucrose. Secondary fixation was performed using 1% osmium tetroxide (Ted Pella, Inc. Redding, California) in cacodylate buffer. Specimens were incubated at 4°C for 1 h, then rinsed with cacodylate buffer and further with distilled water. *En bloc* staining was performed using 1% aqueous uranyl acetate and incubated at 4°C overnight, then rinsed with distilled water. A graded dehydration series was performed using ethanol, transitioned into acetone, and dehydrated tissues were then infiltrated with EMbed 812 resin and polymerized at 60°C overnight. Semithin sections were at a thickness of 1 µm and stained with Toluidine blue to locate the region of interest. The block face was additionally trimmed for the region of interest and sections were cut to a thickness of 75 nm using an ultramicrotome (Ultracut UCT, Leica Microsystems, Germany) and a diamond knife (Diatome, Hatfield PA). Images were acquired with a JEOL JEM 1400 transmission electron microscope (JEOL, Peabody, MA) at 80 kV on a Gatan Rio CMOS camera (Gatan, Inc., Pleasanton, CA).

### 2.5 Mitochondrial isolation from breast tissue

Mitochondria were isolated from chicken breast tissue as previously described (Frezza et al., 2007), with modifications. Briefly, ∼800 mg of chicken breast muscle was thawed in ice-cold PBS/10 mM EDTA. The tissue was then finely minced with ice-cold scissors and washed 2x with PBS/10 mM EDTA. The minced muscle tissues were incubated on ice for 30 min in PBS/10 mM EDTA/0.05% Trypsin. Samples were then centrifuged (10 min, 200 g, 4°C) and supernatant was discarded. The pellet was resuspended in 67 mM sucrose/50 mM Tris-HCl/50 mM KCl/10 mM EDTA/0.2% BSA, pH 7.4 then homogenized via Dounce homogenizer for 10 passes. Samples were then centrifuged (5 min, 200 g, 4°C). The supernatant was transferred to a clean tube, and the process was repeated twice. The supernatant was again transferred to a new tube and centrifuged (10 min, 700 g, 4°C). Supernatant was transferred to a clean tube and centrifuged (10 min, 8,000 g, 4°C). The pellet was resuspended in ice-cold 250mM sucrose/3mM Tris-EGTA/10 mM Tris-HCl, pH 7.4, then centrifuged (10 min, 8,000 g, 4°C). The supernatant was removed by decanting, and the mitochondrial pellet was resuspended in the remaining buffer.

### 2.6 Mitochondrial respiration

Primary myotubes were subject to hypoxia as described above and mitochondrial respiration was measured using the Seahorse XF flux analyzer (Agilent, Santa Clara, CA), as previously described (Dhamad et al., 2021). Basal respiration, ATP production, proton leak, non-mitochondrial oxygen consumption, maximal respiration, and spare respiratory capacity were calculated as previously described (Lassiter et al., 2014; Lassiter et al., 2015). The respiratory capacity of complex I, II, and IV was measured in isolated mitochondria from normal and WB muscle using the protocol of Osto et al. (2020), with 5 µg of mitochondria per well. Mitochondrial respiratory capacity through complex I, II, and IV was calculated as follows:

$$\text{Complex }I:{\text{OCR}}_{\text{NADH}}-{\text{OCR}}_{\text{antimycin}}$$

$$\text{Complex II}:{\text{OCR}}_{\text{succinate}+\text{rotenone}}-{\text{OCR}}_{\text{antimycin}}$$

$$\text{Complex IV}:{\text{OCR}}_{\text{TPMD}+\text{ascorbate}}-{\text{OCR}}_{\text{azide}}$$

### 2.7 RNA extraction and RT-qPCR

Total RNA was extracted using Trizol reagent (Life Technologies, Carlsbad, CA) according to the manufacturer’s protocol, and concentration and quality were determined using the Take3 microvolume plate of the Synergy HTX multimode microplate reader (BioTek, Winooski, VT). cDNA synthesis and qPCR were performed as previously described (Lassiter et al., 2015). Briefly, RNA was reverse transcribed using qScript cDNA Synthesis Supermix (Quanta Biosciences, Gaithersburg, MD), and amplified by qPCR (Applied Biosystems 7,500 Real Time System) with Power-Up Sybr green master mix (Life Technologies, Carlsbad, CA). Relative expression of the target genes was determined using the 2^−ΔΔCT^ method, with normalization to ribosomal 18s gene expression (Schmittgen and Livak, 2008). Oligonucleotide primer sequences specific to chicken are presented in Table 1.

**TABLE 1:** Oligonucleotide qPCR primers.

Gene^a^	Accession number^b^	Primer sequence (5′-3′)	Orientation	Product size, bp
ANT1	NM_204231	GCAGCTGATGTCGGCAAA	For	56
		CAG​TCC​CCG​AGA​CCA​GAG​AA	Rev	
UCP	NM_204107	TGGCAGCGAAGCGTCAT	For	59
		TGG​GAT​GCT​GCG​TCC​TAT​G	Rev	
NFE2L2	NM_205117	AAA​CGA​CAA​CCT​GGC​TGA​AGT​AA	For	59
		TCT​CCG​CTG​GCT​TGG​TTT​C	Rev	
SKI	NM_001039318	GGCCCTGCTGCTTTCTCA	For	75
		AGG​TTC​CGC​TGG​GTC​TTT​G	Rev	
DNM1	XM_015279546	GAACTTTCGCCCCGATGA	For	57
		TGG​ACC​ATC​TGA​AGC​AGA​GCT​T	Rev	
MFN1	NM_001012931	CGG​TGG​TTT​TGA​GCC​CAT​T	For	57
		GAA​GCC​TGG​CAC​CCA​AAT​C	Rev	
MFN2	XM_040689232	ATG​TGC​CTG​TGA​CAC​GTT​CAC	For	63
		TCG​AGT​GTC​AGG​CAG​CTT​CTT	Rev	
OMA1	XM_422503	TCA​CTA​TGA​TTT​GGG​CCA​TCT​G	For	59
		GATCCGCTGGCCAACAAC	Rev	
OPA1	NM_001039309	CCC​AAG​CAG​GAT​CCA​ACA​A	For	73
		AAC​AAC​TGC​AAA​GTA​ACC​CAA​AGC	Rev	
mtDNA	X52392	ACA​CCT​GCG​TTG​CGT​CCT​A	For	58
		ACG​CAA​ACC​GTC​TCA​TCG​A	Rev	
PGC1α	NM_001006457	GAG​GAT​GGA​TTG​CCT​TCA​TTT​G	For	62
		GCG​TCA​TGT​TCA​TTG​GTC​ACA	Rev	
PGC1β	XM_040647119	TTGCCGGCATTGGTTTCT	For	66
		CACGGGAAGCCACAGGAA	Rev	
SSBP1	NM_001278007	CAC​AGA​CAG​GTG​ATA​TCA​GTC​AGA​AG	For	65
		GAG​GCC​TGG​TCT​GAA​GAC​AGA	Rev	
PPARα	NM_001001464	CAA​ACC​AAC​CAT​CCT​GAC​GAT	For	64
		GGA​GGT​CAG​CCA​TTT​TTT​GGA	Rev	
PPARγ	NM_001001460	CAC​TGC​AGG​AAC​AGA​ACA​AAG​AA	For	67
		TCC​ACA​GAG​CGA​AAC​TGA​CAT​C	Rev	
ITPR1	XM_046925976	TCC​GTG​TAC​GTT​TAG​TTC​ATC​TTG​TAA	For	116
		CGGCGTGTGCAAACAGT	Rev	
ITPR2	XM_040657966	GGA​AGT​TTT​GGA​TGT​GGT​CAT​TAC​T	For	90
		ACT​CCG​AAT​ATC​TGA​GCC​AAA​AT	Rev	
ITPR3	XM_040691640	TGC​ACG​CCA​GCA​ACT​ATG​AG	For	87
		GGT​TGA​TTT​TCC​AGC​TGG​TGT​T	Rev	
INF2	XM_040672115	AAA​CCT​TGC​CTG​CGG​AGA​T	For	61
		TGC​GGA​TCC​TTA​ATG​CTC​TTC	Rev	
FUNDC1	NM_001276363	CGCACCGCCCCAGAA	For	61
		ATT​CCG​TTA​GGT​CCA​ACA​CTT​CA	Rev	
TOMM20	XM_423972	TCG​GCT​ACT​GCA​TCT​ACT​TCG​A	For	62
		CAG​CCG​GTT​CTT​GAA​ATT​CG	Rev	
VDAC1	NM_001033869	GGC​TGC​GAC​ATG​GAT​TTT​G	For	55
		GCA​CCA​GGG​CTC​CAC​GTA​T	Rev	
SPIRE1	XM_040664833	AAG​TGA​TCG​GGG​ATT​TAC​GAA	For	60
		TGT​ATT​GAC​GCT​CTT​GGA​CTT​TCT	Rev	
18s	AF173612	TCC​CCT​CCC​GTT​ACT​TGG​AT	For	60
		GCGCTCGTCGGCATGTA	Rev	

^a^ ANT1, adenine nucleotide translocator 1; UCP, uncoupling protein; NFE2L2, nuclear factor erythroid 2-related factor 2; SKI, nuclear sarcoma viral oncogene homolog; DNM1, dynamin-related protein 1; MFN1, mitofusin 1; MFN2, mitofusin 2; OMA1, OMA1 zinc metallopeptidase; OPA1, OPA1 mitochondrial dynamin like GTPase; mtDNA, mitochondrial DNA; PGC1α, peroxisome proliferator-activated receptor gamma coactivator 1-alpha; PGC1β, peroxisome proliferator-activated receptor gamma coactivator 1-beta; SSBP1, single stranded DNA binding protein 1; PPARα, peroxisome proliferator activated receptor alpha; PPARγ, peroxisome proliferator activated receptor gamma; ITPR1, inositol 1,4,5-trisphosphate receptor type 1; ITPR2, inositol 1,4,5-trisphosphate receptor type 2; ITPR3, inositol 1,4,5-trisphosphate receptor type 3; INF2, inverted formin 2; FUNDC1, FUN14 domain-containing protein 1; TOMM20, translocase of outer mitochondrial membrane 20; VDAC1, voltage dependent anion channel 1; SPIRE1, spire type actin nucleation factor 1.

^b^ Accession number refers to GenBank (National Center for Biotechnology Information – NCBI).

### 2.8 Western blot

Western blot was performed as previously described (Lassiter et al., 2015). Briefly, muscle tissue, isolated mitochondria, and primary cells were homogenized in lysis buffer containing protease- and phosphatase-inhibitors. Protein concentrations were determined via Bradford assay kit (Bio-Rad, Hercules, CA) and the Synergy HTX multimode microplate reader (BioTek, Winooski, VT). Proteins were separated on 4%–12% gradient Bis-Tris gels (Life Technologies, Carlsbad, CA), and transferred to PVDF membranes. Membranes were blocked with 5% non-fat milk in TBS-T for 1 h at room temperature, then incubated with primary antibodies overnight at 4°C. Primary antibodies used were rabbit anti-ANT1 (1:1,000, PA1-85116, ThermoFisher Scientific, Waltham, MA), rabbit anti-INF2 (1:1,000, A303-427A, Bethyl Laboratories, Montgomery, TX), rabbit anti-ITPR2 (1:1,000, A19320, ABClonal, Woburn, MA), rabbit anti-MFN1 (1:1,000, ab104274, Abcam, Boston. MA), rabbit anti-MFN2 (1:1,000, 12186-1-AP, Proteintech, Rosemont, IL), rabbit anti-OMA1 (1:1,000, ab104316, Abcam, Boston, MA), rabbit anti-OPA1 (1:1,000, A9833, ABClonal, Woburn, MA), rabbit anti VDAC1 (1:1,000, 4,866, Cell Signalling, Danvers, MA), and OXPHOS antibody cocktail (1:1,000, ab110413, Abcam, Boston. MA). Rabbit anti-GAPDH (1:1,000, NB300-327, Novus Biologicals, Centennial, CO) was used as a loading control, with representative blots shown. HRP-conjugated secondary antibodies (goat anti-rabbit IgG #7074 and rabbit anti-mouse IgG #7076, Cell Signaling, Danvers, MA) were used at 1:5,000 dilution for 1 h at room temperature. The signal was visualized by chemiluminescence (Super ECL, ABP Biosciences, North Potomac, MD) and captured by FluorChem M MultiFluor System (ProteinSimple, Santa Clara, CA). Image acquisition and analysis were performed with AlphaView software (version 3.4.0.0, ProteinSimple, Santa Clara, CA).

### 2.9 Calcium assay

Total calcium concentration in muscle tissue and primary myotubes was measured by a Calcium Assay Kit (#701220, Cayman Chemical, Ann Arbor, MI) according to manufacturer’s recommendations.

### 2.10 ATP synthase enzyme activity and ATP assay

ATP Synthase Enzyme activity was measured in isolated mitochondria from normal and WB tissues and primary myotubes using the ATP synthase Enzyme Activity Microplate Assay Kit (ab109714, Abcam, Boston, MA) according to manufacturer’s recommendations. Briefly, 5ug of protein was plated in duplicate into a 96 well plate. The activity of the ATP synthase enzyme is coupled to the molar conversion of NADH to NAD^+^ and is measured as a decrease in absorbance at OD 340 nm. The activity rate is expressed as the change in absorbance at 340 nm/min/amount of sample. The rate was calculated over the linear phase of incubation.

ATP levels were measured using the ATP Assay Kit (ab83355, Abcam, Waltham, MA). Muscle tissues from normal and WB-affected birds were homogenized in ice cold 2 N perchloric acid and kept on ice for 30 min. Tissue samples were centrifuged at 13,000 g for 2 min, and supernatant collected. Supernatant was diluted 1:5 with ATP assay buffer, and excess perchloric acid precipitated with 2 M KOH. Samples were again centrifuged at 13,000 g for 2 min, and the supernatant used for the ATP assay, according to manufacturer’s protocol.

### 2.11 Statistical analyses

Data were analyzed by Student “t” test using Graph Pad Prism software (version 9.03 for Windows, Graph Pad Software, La Jolla California, United States). All data are expressed as the mean ± SEM and were considered statistically significant at a *P* value ≤0.05.

## 3 Results

### 3.1 Calcium concentration is higher in WB muscle and hypoxic primary myoblasts

**FIGURE 1:**
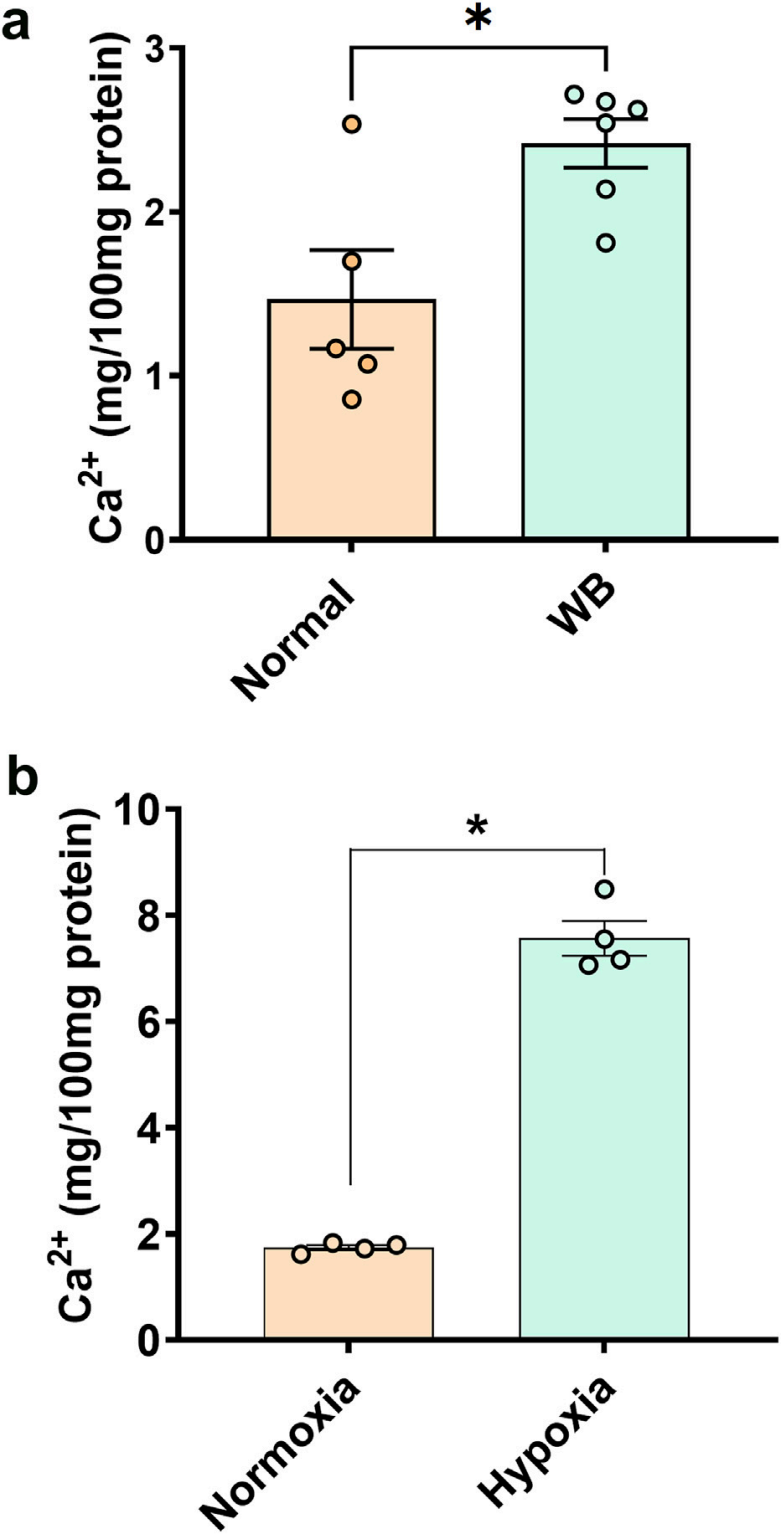
Total calcium concentration in chicken muscle **(A)** and primary myoblasts **(B)**. Data are expressed as means ± SEM (n = 4–6/group). *Significantly different at *P* < 0.05.

Total calcium concentration in WB tissue extracts was significantly higher (*P* = 0.0079) than normal controls (Figure 1A). Similarly, chicken primary myotubes exposed to hypoxic conditions had higher calcium than their normoxic controls (*P* < 0.0001, Figure 1B).

### 3.2 Mitochondrial morphology is altered in WB muscle

**FIGURE 2:**
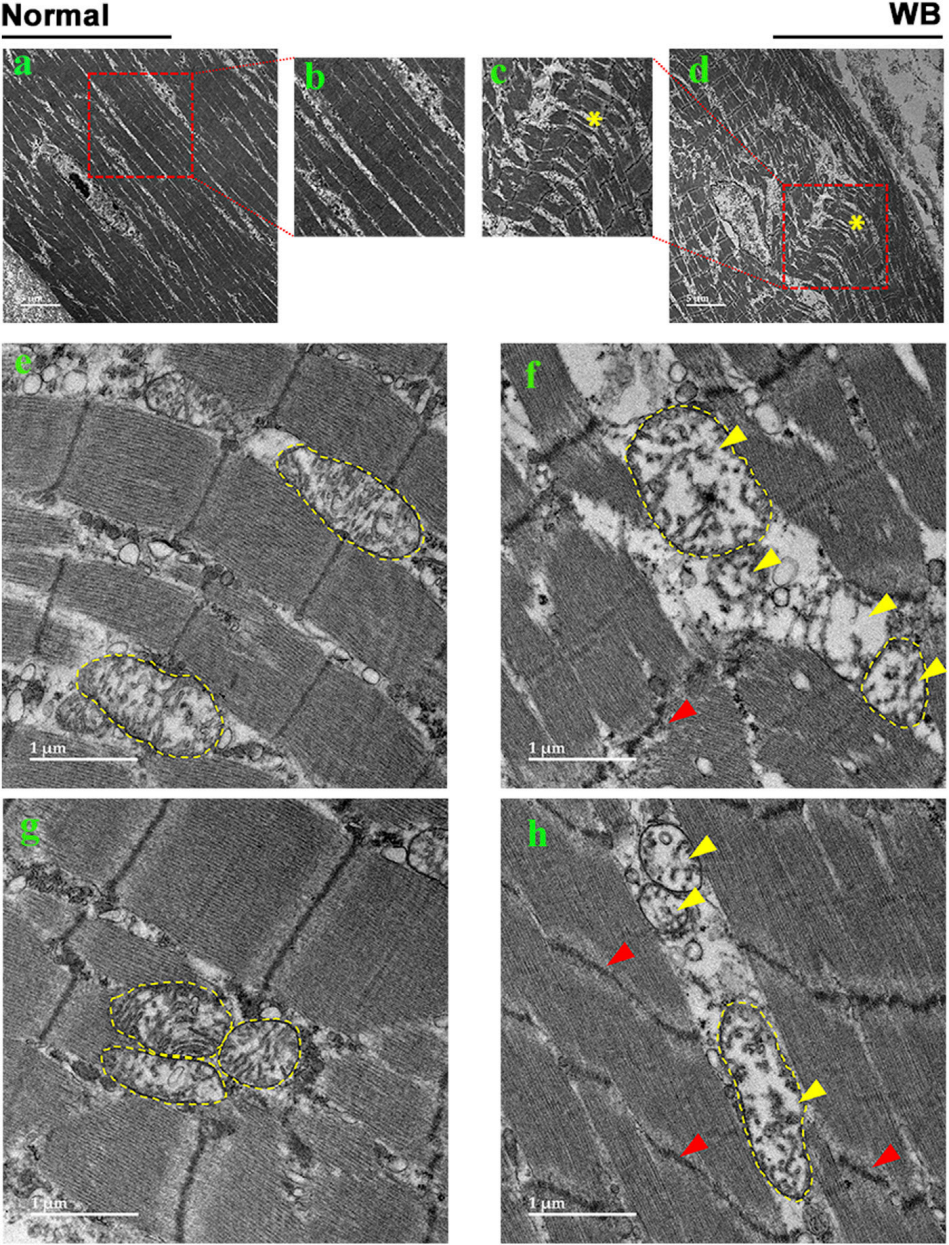
Electron microscopy images of normal **(A, B, E, G)** and woody breast **(C, D, F, H)**. Yellow arrows - mitochondria with degraded cristae. Red arrows - Z-line streaming. “*” – wave-like pattern in myofibers.

As compared to normal tissue, WB muscle showed distorted myofiber structure (Figures 2A–D). The linearity of the myofibrils was disrupted and formed wave-like patterns in WB (Figures 2C, D, asterisk). Although myofiber separation was evident in both normal and WB muscle, the degree of separation was much higher in WB. In addition, Z line streaming was evident (Figures 2F, H, red arrows). Swollen and/or elongated mitochondria (0.72 ± 0.1 vs. 0.3 ± 0.03 in WB and normal muscle, respectively, *P* < 0.05) with indistinct cristae structure were observed in WB-affected muscle (Figures 2F, H, yellow arrows) as compared to normal muscle (Figures 2E, G).

### 3.3 Disruption of mitochondrial network genes in WB muscle and hypoxic primary myoblasts

#### 3.3.1 Mitochondrial function

**FIGURE 3:**
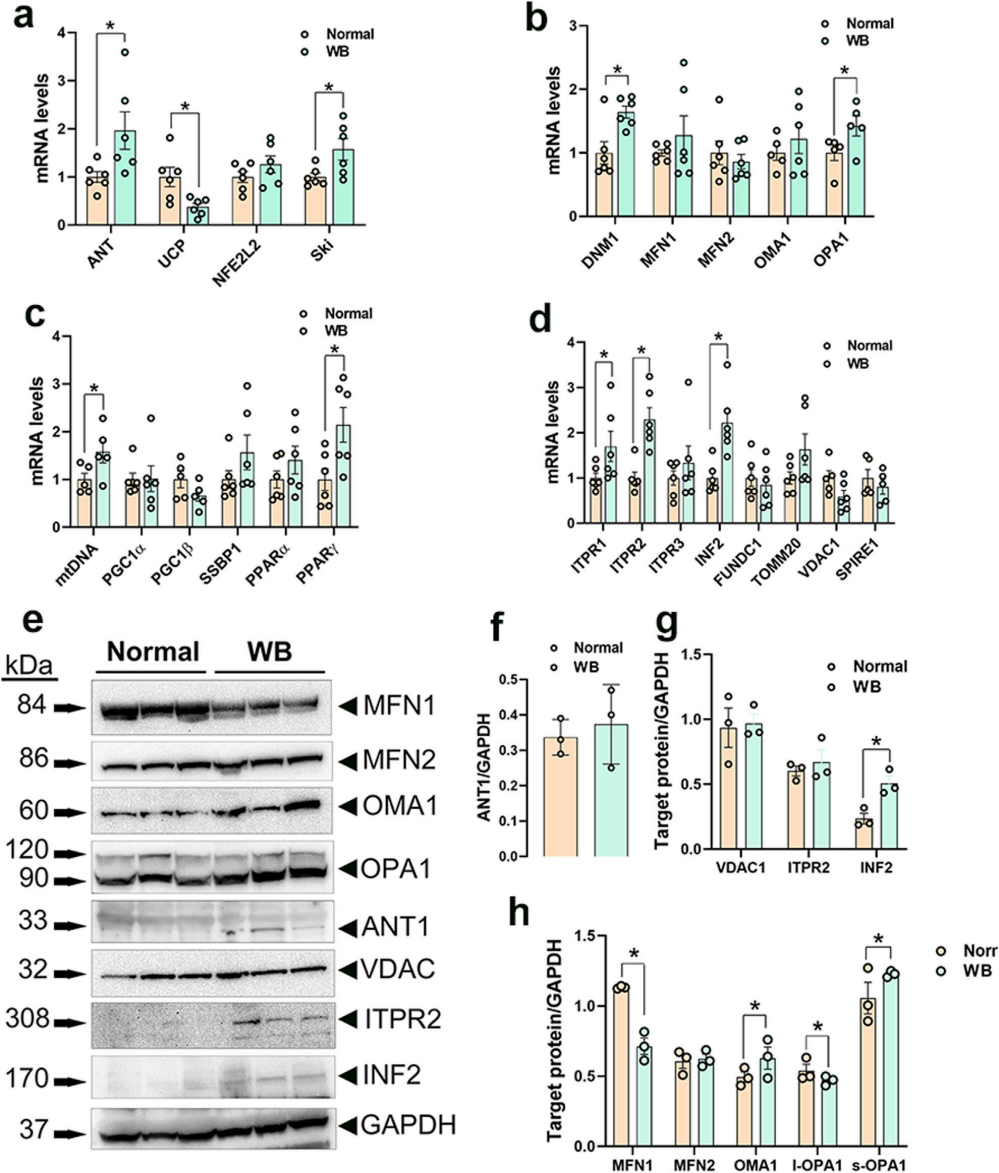
Mitochondrial network-related gene and protein expression in chicken breast muscle. Gene expression for mitochondrial function **(A)**, dynamics **(B)**, biogenesis **(C)**, and tethering **(D)** was determined by qPCR. Protein levels were determined by Western blot **(E–H)**. Data are expressed as means ± SEM (n = 6/group). *Significantly different at *P* < 0.05. ANT1, adenine nucleotide translocase; DNM1, dynamin-related protein 1; FUNDC1, FUN14 domain containing 1; INF2, inverted formin 2; ITPR1, inositol 1,4,5-trisphosphate receptor type 1; ITPR2, inositol 1,4,5-trisphosphate receptor type 2; ITPR3, inositol 1,4,5-trisphosphate receptor type 3; MFN1, mitofusin 1; MFN2, mitofusin 2; mtDNA, mitochondrial DNA; NFE2L2, nuclear factor erythroid 2-related factor 2; PGC1α, peroxisome proliferator-activated receptor gamma coactivator 1 alpha; PGC1β, peroxisome proliferator-activated receptor gamma coactivator 1 beta; PPARα, peroxisome proliferator activated receptor alpha; PPARγ, peroxisome proliferator activated receptor gamma; OMA1, OMA1 zinc metallopeptidase; OPA1, OPA1 mitochondrial dynamin like GTPase; SKI, nuclear sarcoma viral oncogene homolog; SPIRE1, spire type actin nucleation factor 1; SSBP1, mitochondrial single-stranded DNA binding protein 1; TOMM20, translocase of outer mitochondrial membrane 20; VDAC1, voltage dependent anion channel 1; UCP, uncoupling protein.

**FIGURE 4:**
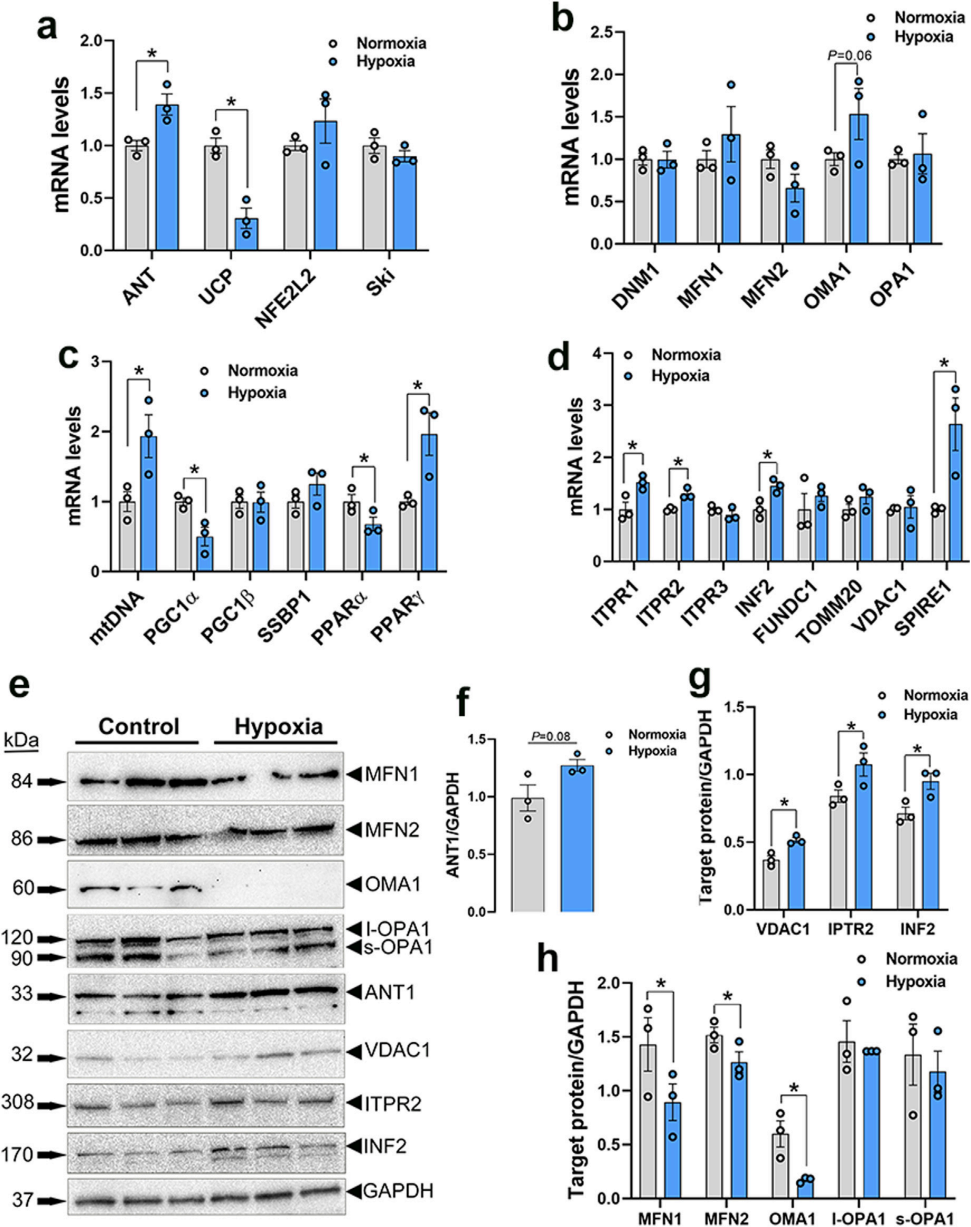
Mitochondrial network-related gene and protein expression in primary myoblasts. Gene expression for mitochondrial function **(A)**, dynamics **(B)**, biogenesis **(C)**, and tethering **(D)** was determined by qPCR. Protein levels were determined by Western blot **(E–H)**. Data are expressed as means ± SEM (n = 6/group), representative blots are shown. *Significantly different at *P* < 0.05. ANT1, adenine nucleotide translocase; DNM1, dynamin-related protein 1; FUNDC1, FUN14 domain containing 1; INF2, inverted formin 2; ITPR1, inositol 1,4,5-trisphosphate receptor type 1; ITPR2, inositol 1,4,5-trisphosphate receptor type 2; ITPR3, inositol 1,4,5-trisphosphate receptor type 3; MFN1, mitofusin 1; MFN2, mitofusin 2; mtDNA, mitochondrial DNA; NFE2L2, nuclear factor erythroid 2-related factor 2; PGC1α, peroxisome proliferator-activated receptor gamma coactivator 1 alpha; PGC1β, peroxisome proliferator-activated receptor gamma coactivator 1 beta; PPARα, peroxisome proliferator activated receptor alpha; PPARγ, peroxisome proliferator activated receptor gamma; OMA1, OMA1 zinc metallopeptidase; OPA1, OPA1 mitochondrial dynamin like GTPase; SKI, nuclear sarcoma viral oncogene homolog; SPIRE1, spire type actin nucleation factor 1; SSBP1, mitochondrial single-stranded DNA binding protein 1; TOMM20, translocase of outer mitochondrial membrane 20; VDAC1, voltage dependent anion channel 1; UCP, uncoupling protein.

Among the genes involved in mitochondrial function, adenine nucleotide translocase 1 (ANT1) expression was significantly increased in both WB muscle (*P* = 0.0264, Figure 3A) and hypoxic primary myoblasts (*P* = 0.0128, Figure 4A) compared to normal breast muscle and normoxic cells, respectively. Sloan-Kettering Institute (SKI) proto-oncogene (Ski) mRNA abundance was upregulated in WB (*P* = 0.0238, Figure 3A) compared to normal breast muscle, but remained unchanged in both normoxic and hypoxic myoblasts (Figure 4A). Avian uncoupling protein (av-UCP) gene expression was significantly downregulated in both WB tissue and hypoxic myoblast compared to normal breast muscle and normoxic cells, respectively (Figures 3A, 4A). Nuclear factor erythroid-derived 2-like 2 (NFE2L2) mRNA abundances and ANT1 protein levels did not change between all tissues and cells (Figures 3A, E).

#### 3.3.2 Mitochondrial dynamics

Dynamin-related protein 1 (*DNM1*, *P* = 0.0056) and optic atrophy type 1 (*OPA1*, *P* = 0.0318) gene expressions were significantly upregulated in WB as compared to normal muscle (Figure 3B), and OMA1 zinc metallopeptidase (OMA1) mRNA levels were significantly increased in hypoxic compared to normoxic cells (Figure 4B). The other mitochondrial dynamics-related genes were not affected (Figures 3B, 4B). At the protein level, only mitofusin 1 (MFN1) was significantly decreased in WB compared to normal muscle, however both MFN1 and MFN2 proteins were significantly reduced in hypoxic compared to normoxic cells (Figures 3E, H, 4E, H). The expression of OMA1 protein was significantly increased in WB compared to normal muscle (Figures 3E, H), but it was significantly decreased in hypoxic compared to normoxic cells (Figures 4E, H). Protein levels of l-OPA1 were significantly diminished and that of s-OPA1 were significantly induced only in WB compared to normal muscle (Figures 3E, H), but not in hypoxic cells (Figures 4E, H).

#### 3.3.3 Mitochondrial biogenesis

Mitochondrial DNA (mtDNA)-D loop and peroxisome proliferator activated receptor gamma (*PPARγ*) gene expression were both significantly upregulated in WB (Figure 3C) and hypoxic cells (Figure 4C) as compared to normal muscles and normoxic cells, respectively. Peroxisome proliferator-activated receptor gamma coactivator 1 alpha (*PGC1α*, *P* = 0.0129) and peroxisome proliferator-activated receptor alpha (*PPARα*, *P* = 0.0491) gene expressions were downregulated in the hypoxic compared to normoxic cells (Figure 4C) but not in WB muscles (Figure 3C). The expression of PGC1β and single stranded DNA binding protein 1 (SSBP1) genes did not differ between all tissue and cell groups (Figures 3C, 4C).

#### 3.3.4 Mitochondrial-endoplasmic reticulum tethering

The expression of inositol 1,4,5-trisphosphate receptor type 1 and 2 (*ITPR1 and ITPR2*) and inverted formin 2 (IFN 2) genes was significantly upregulated in both WB and hypoxic cells compared to normal breast tissue and normoxic cells, respectively (Figures 3D, 4D). The expression of spire type actin nucleation factor 1 (SPIRE1) was significantly upregulated only in hypoxic cells compared to normoxic ones (Figure 4D), but not in WB muscles (Figure 3D). The expression of voltage dependent anion channel 1 (*VDAC1*), FUN14 domain containing 1 (FUNDC1), and translocase of outer mitochondrial membrane 20 (TOMM20) genes was not affected neither by the WB myopathy nor by the hypoxia exposure of primary myoblasts (Figures 3D, 4D). At the protein levels, VDAC1, ITPR2, and INF2 were all significantly increased in hypoxic compared to normoxic cells (Figures 4E, G), however only INF2 protein levels were significantly induced in WB compared to normal breast tissues (Figures 3E, G).

#### 3.3.5 Altered mitochondrial bioenergetics in WB muscle and hypoxic primary myoblasts

**FIGURE 5:**
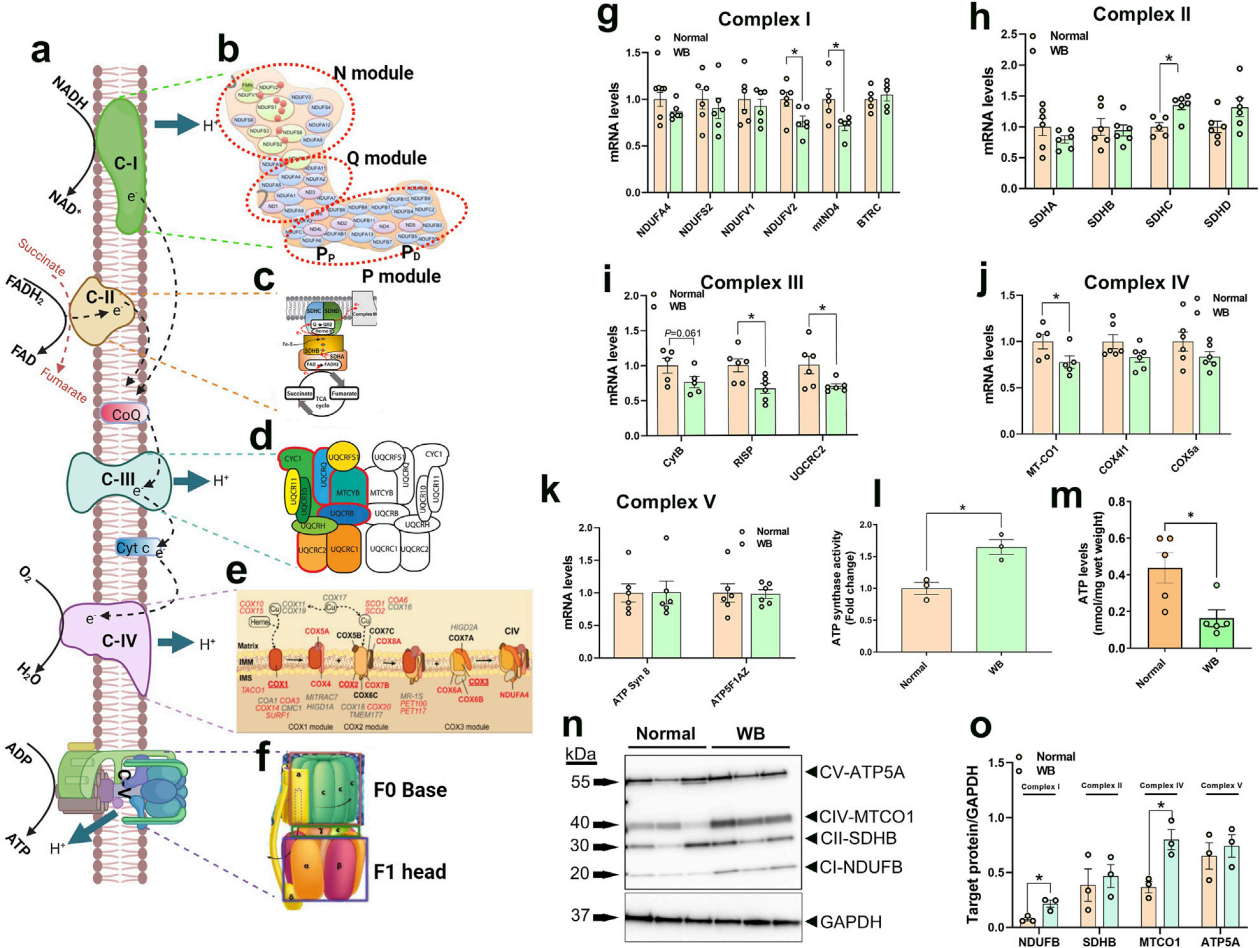
Expression profile of genes associated with mitochondrial ETC in chicken breast muscle. A schematic illustration of ETC and its complexes [I to V, **(A–F)**] (not for scale). Gene expression for complex I **(G)**, II **(H)**, III **(I)**, IV **(J)**, and V **(K)** was determined by qPCR. ATP synthase activity was determined in muscle isolated mitochondria by Enzyme Activity Microplate Assay Kit **(L)** and ATP levels were measured by ATP assay kit **(M)**. Protein levels were determined by immunoblot **(N, O)** and representative blots are shown. Data are expressed as means ± SEM (n = 6/group). *Significantly different at *P* < 0.05. ATP5FAZ, ATP synthase F1 subunit alpha Z chromosome; ATPSyn8, ATP Synthase F0 subunit 8; COX4I1, cytochrome C oxidase subunit 4I1; COX5A, cytochrome C oxidase subunit 5A; CytB, mitochondrially encoded cytochrome B; MT-CO1, mitochondrially encoded cytochrome C oxidase I; NDUFA4, NADH dehydrogenase (Ubiquinone) 1 alpha subcomplex subunit 4; NDUFS2, NADH:ubiquinone oxidoreductase core subunit S2; NDUFV1, NADH:ubiquinone oxidoreductase core subunit V1; NDUFV1, NADH:ubiquinone oxidoreductase core subunit V2; mtND4, mitochondrially encoded NADH:ubiquinone oxidoreductase core subunit 4; RISP, ubiquinol-cytochrome C reductase, Rieske iron-sulfur polypeptide 1; SDHA, succinate dehydrogenase complex flavoprotein subunit A; SDHB, succinate dehydrogenase complex iron sulfur subunit B; SDHC, succinate dehydrogenase complex subunit C; SDHD, succinate dehydrogenase complex subunit D; UQCRC2, ubiquinol-cytochrome C reductase core protein 2. Figures 5B–F were modified from (Saada, 2013) **(B)** (Moosavi et al., 2019) **(C)** (Tucker et al., 2013) **(D)** (Hock et al., 2020) **(E)**, and (Ahern and Rajagopal, 2024) **(F)**, with permission from Springer Nature license (#5915970048082 and 5916040393036).

**FIGURE 6:**
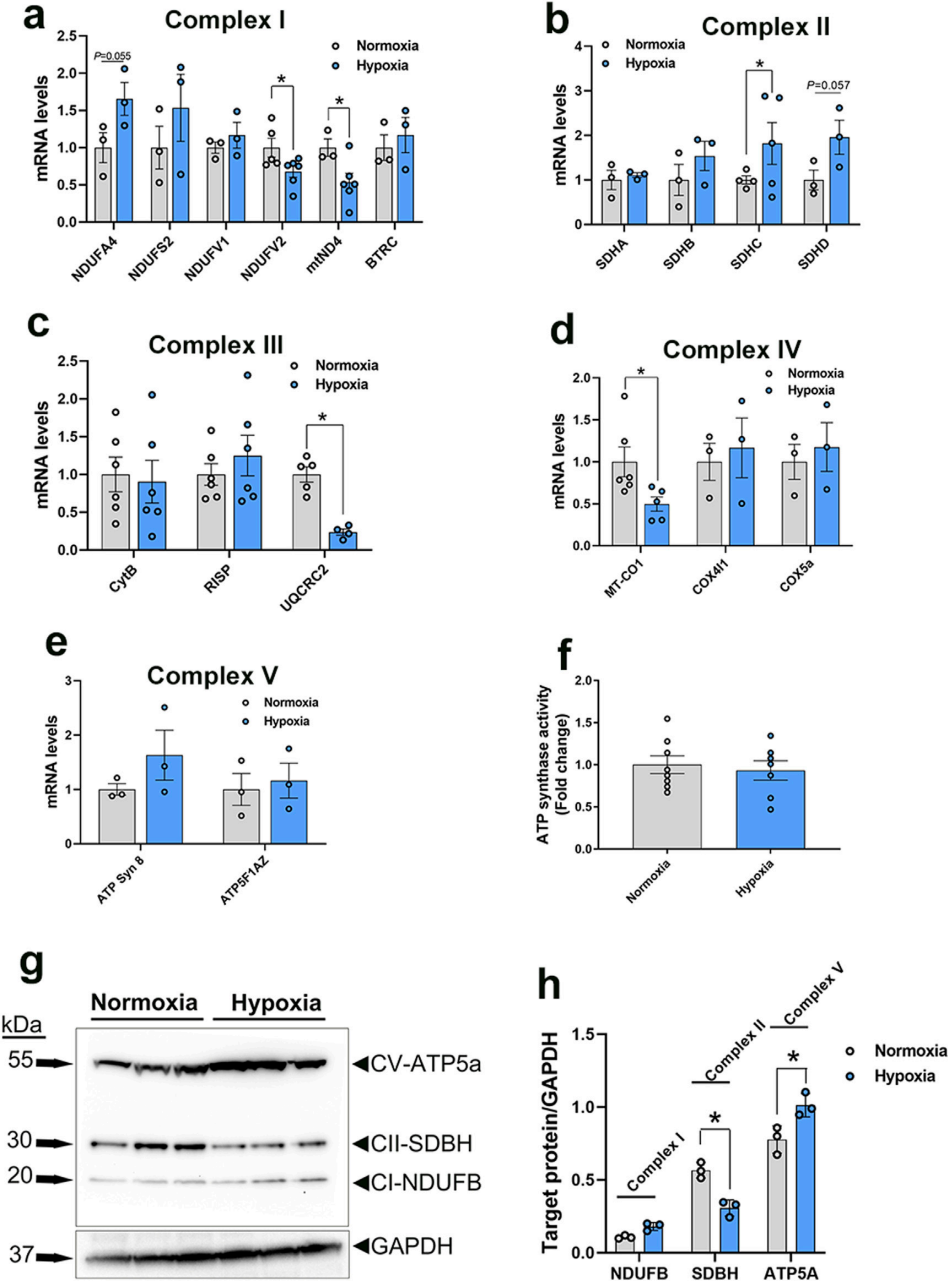
Expression profile of genes associated with mitochondrial ETC in primary myoblasts. Gene expression for complex I **(A)**, II **(B)**, III **(C)**, IV **(D)**, and V **(E)** was determined by qPCR. ATP synthase activity was determined in cells by Enzyme Activity Microplate Assay Kit **(F)**. Protein levels were determined by immunoblot **(G, H)** and representative blots are shown. Data are expressed as means ± SEM (n = 6/group). *Significantly different at P < 0.05. ATP5FAZ, ATP synthase F1 subunit alpha Z chromosome; ATPSyn8, ATP Synthase F0 subunit 8; COX4I1, cytochrome C oxidase subunit 4I1; COX5A, cytochrome C oxidase subunit 5A; CytB, mitochondrially encoded cytochrome B; MT-CO1, mitochondrially encoded cytochrome C oxidase I; NDUFA4, NADH dehydrogenase (Ubiquinone) 1 alpha subcomplex subunit 4; NDUFS2, NADH:ubiquinone oxidoreductase core subunit S2; NDUFV1, NADH:ubiquinone oxidoreductase core subunit V1; NDUFV1, NADH:ubiquinone oxidoreductase core subunit V2; mtND4, mitochondrially encoded NADH:ubiquinone oxidoreductase core subunit 4; RISP, ubiquinol-cytochrome C reductase, Rieske iron-sulfur polypeptide 1; SDHA, succinate dehydrogenase complex flavoprotein subunit A; SDHB, succinate dehydrogenase complex iron sulfur subunit B; SDHC, succinate dehydrogenase complex subunit C; SDHD, succinate dehydrogenase complex subunit D; UQCRC2, ubiquinol-cytochrome C reductase core protein 2.

A schematic illustration of mitochondrial complexes is presented in Figure 5A. Gene expression of components of Complex I was altered in WB-affected muscles. The expression of the NADH-ubiquinone oxidoreductase core subunit V2 (NDUFV2), belonging to N-module, and NADH dehydrogenase 4 (mtND4), belonging to P-module, was downregulated in WB-affected muscles (Figure 5G) and in hypoxic myoblasts (Figure 6A) compared to normal muscles and normoxic cells, respectively. The expression of NADH-ubiquinone oxidoreductase MLRQ subunit (NDUFA4), NADH dehydrogenase [Ubiquinone] iron-sulfur protein (NDUFS2), NADH dehydrogenase [Ubiquinone] flavoprotein (NDUFV1), and beta-transducin repeat containing E3 ubiquitin protein ligase (BTRC) genes remained unchanged between WB-affected and healthy muscles (Figure 5G).

In the mitochondrial complex II, only succinate dehydrogenase complex subunit C (SDHC) gene expression was upregulated, but not that of succinate dehydrogenase complex flavoprotein subunit A (SDHA), succinate dehydrogenase complex iron sulfur subunit B (SDHB), and succinate dehydrogenase complex subunit D (SDHD), in WB-affected and hypoxic cells compared to normal muscles and normoxic cells, respectively (Figures 5H, 6B).

The expression of the complex III-associated genes, rieske iron-sulfur protein (RISP) and ubiquinol-cytochrome C reductase core protein 2 (UQCRC2) was significantly downregulated in WB compared to healthy muscles (Figure 5I), however only UQCRC2 gene expression was downregulated in hypoxic compared to normoxic cells (Figure 6C). The expression of cytochrome B subunit (CytB) remained unchanged between all muscle and cell groups (Figures 5I, 6C).

In complex IV, cytochrome C oxidase subunit I (MT-CO1) mRNA abundances, but not that of cytochrome C oxidase subunit 4 isoform 1 (COX4I1) and cytochrome C oxidase subunit 5 A (COX5a), were significantly decreased in WB and hypoxic myoblasts compared to healthy muscles and normoxic cells, respectively (Figures 5J, 6D).

Although the expression of complex V-associated genes, ATP synthase 8 (ATP8) and ATP synthase F1 subunit alpha Z chromosome (ATP5F1AZ), was not affected (Figure 5K). Although ATP synthase activity was significantly increased (Figure 5L), ATP levels were significantly decreased in WB compared to healthy muscles (Figure 5M). Neither ATP8, ATP5F1AZ nor ATP synthase activity was affected in hypoxic cells (Figures 6E, F).

**FIGURE 7:**
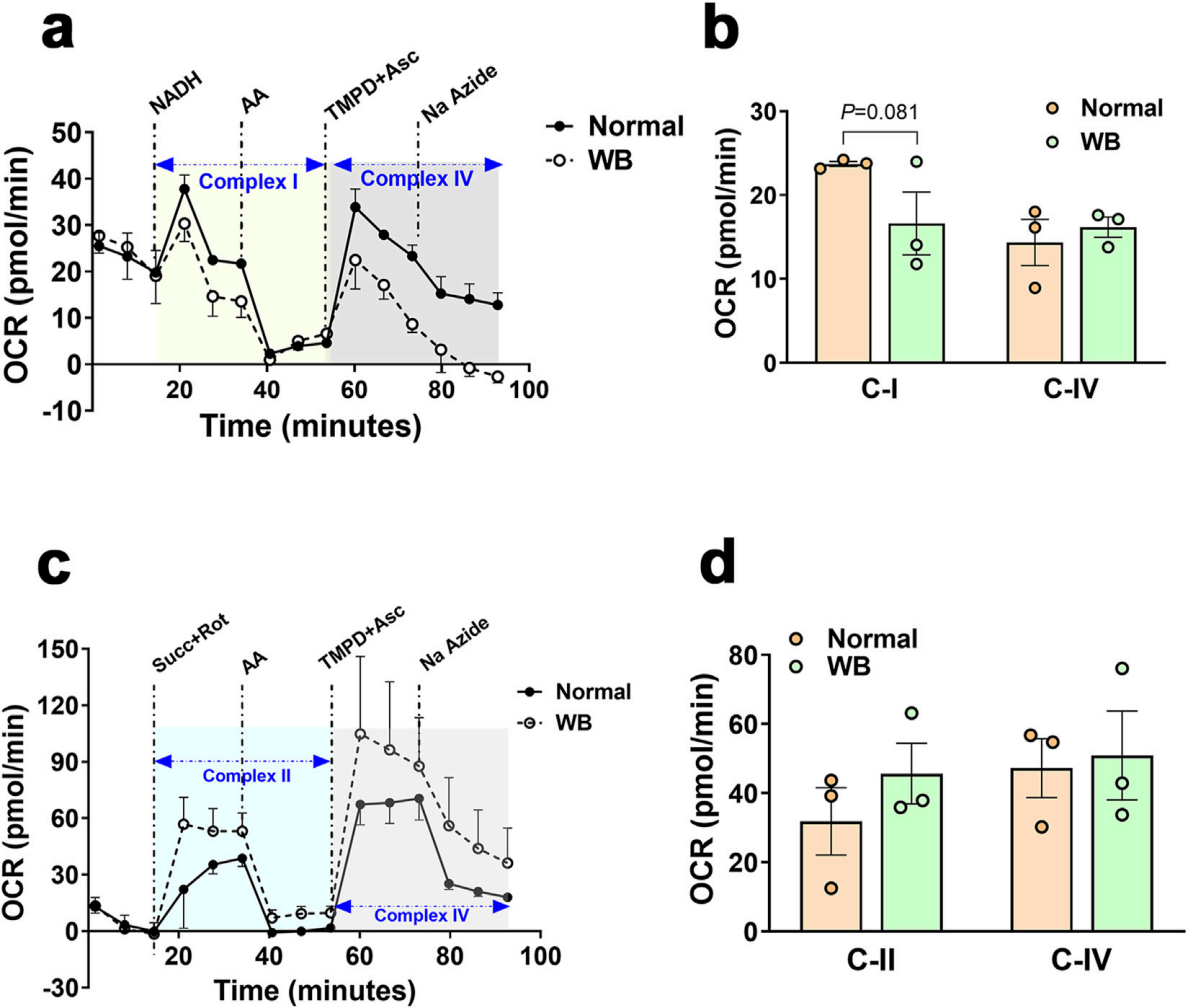
Respirometry measurement of mitochondrial complex I, II, and IV in chicken breast muscle. The oxygen consumption rate (OCR) was measured in mitochondria isolated from breast muscles treated with various selective (un)coupler reagents using seahorse XF analyzer following the protocol of (Osto et al., 2020). Complex I and IV **(A, B)** and complex II and IV **(C, D)**. Data are mean ± SEM (n = 6). AA, antimycin; Asc, ascorbic acid; Na azide, sodium azide; NADH, Rot, rotenone; Succ, succinate; TMPD, N1,N1,N1,N1-tetramethyl-1,4-phenylene diamine; WB, woody breast.

Immunoblot measurements of OXPHOS proteins indicated that NDUFB (complex I) and MT-CO1 (complex IV) protein levels were significantly increased in WB compared to normal muscles (Figures 5N, O), and SDHB (complex II) and ATP5A (complex V) protein levels were increased in hypoxic compared to normoxic cells (Figures 6G, H). In mitochondria isolated from breast muscle, complex I activity tended to be lower in WB as compared to normal muscles (*P* = 0.081, Figures 7A, B). There were no significant differences in complex II or complex IV (*P* > 0.05, Figures 7C, D).

**FIGURE 8:**
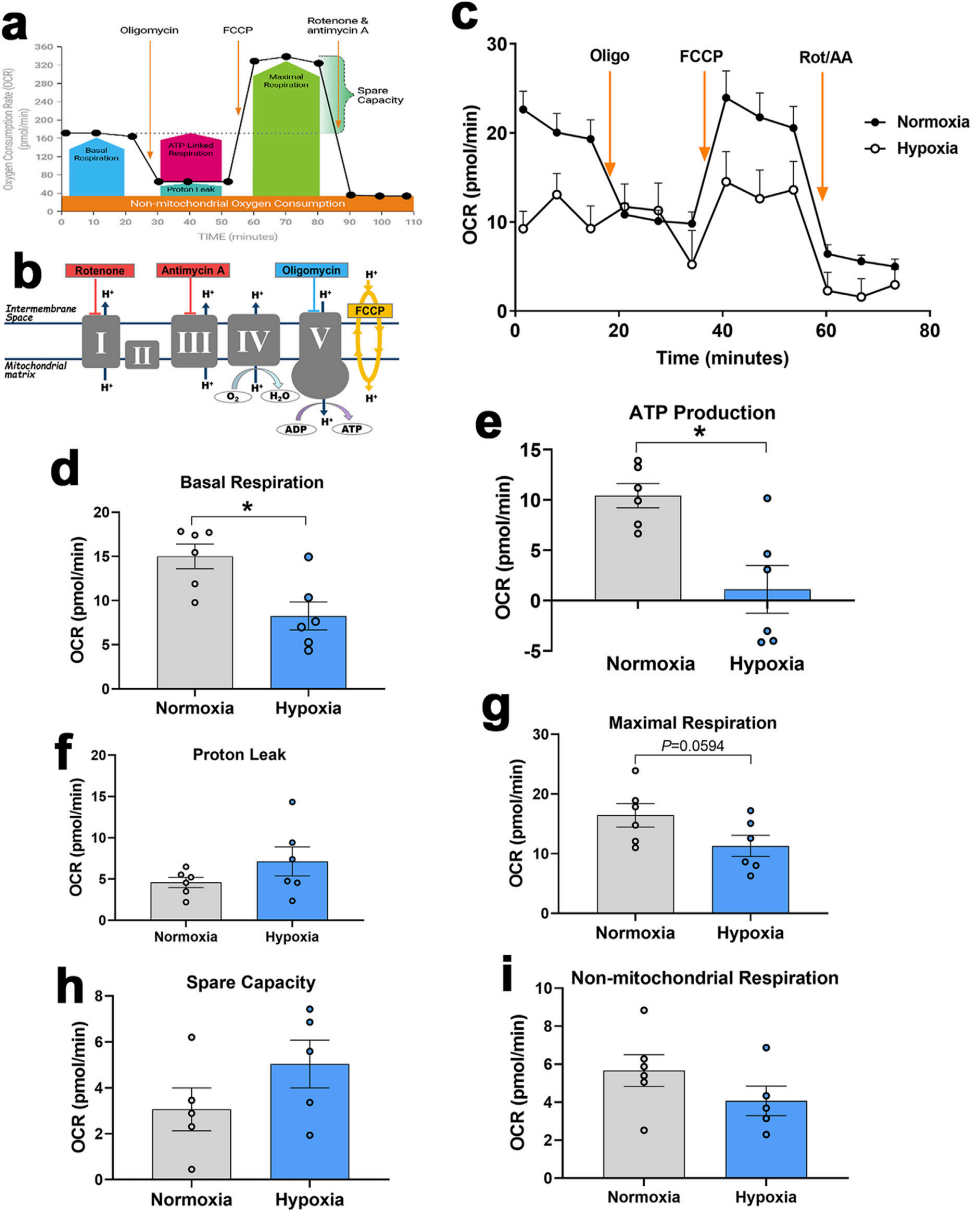
Measurement of mitochondrial respiration in primary myoblasts using Seahorse XF analyzer. Respirometry and OCR were measured in primary myoblasts exposed to normoxia or hypoxia followed by consecutive injections of various selective (un)couplers. Typical OCR patterns from Agilent **(A, B)**, OCR in primary myoblasts **(C)**, basal respiration **(D)**, ATP production **(E)**, proton leak **(F)**, Maximal respiration **(G)**, spare capacity **(H)**, and non-mitochondrial respiration **(I)**. **(A, B)** were obtained from Agilent user guide (kit 103015-100) with permission from Agilent Technologies, Inc. AA, antimycin; FCCP, carbonyl cyanide-4-(trifluoromethoxy)phenylhydrazone; OCR, oxygen consumption rate; Oligo, oligomycin; Rot, rotenone.

Seahorse XF analyzer showed that hypoxic primary myotubes had significantly lower basal respiration (*P* = 0.0057, Figures 8A–D) and ATP production (*P* = 0.0033, Figure 8E) as compared to normoxic cells. There were no significant differences in proton leak, maximal respiration, spare capacity, or non-mitochondrial respiration (Figures 8F–I).

## 4 Discussion

Woody breast is a muscle myopathy that adversely impacts the poultry industry through decreased meat quality and increased condemnations, resulting in significant welfare concerns and heavy economic losses (Kuttappan et al., 2016). Several lines of research have indicated local hypoxia as a concurrent condition (Greene et al., 2019; Emami et al., 2021; Zhang et al., 2024), however the cellular and molecular mechanisms by which hypoxia causes the myopathy are still not completely understood. As mitochondria are the primary consumers of oxygen within the cell and the powerhouse providing the necessary energy for cellular homeostasis and functions, we sought to assess mitochondrial function, biogenesis, dynamics, and bioenergetics within the WB-affected muscles and hypoxic primary myoblasts.

As a hallmark of many muscle disorders, mitochondrial dysfunction presents in multiple ways, including defective OXPHOS, Ca^2+^ imbalances, mtDNA mutations, and structural defects, leading to altered ATP production, decreased mitochondrial respiration, and increased ROS (Chen et al., 2022).

Here, transmission electron microscopy analysis of WB-affected tissues revealed an extremely disordered muscle structure with multiple defects in myofibers and organellar architecture. The wave-like shape of myofibers observed in WB muscle resembles that of human working muscle during exercise and restricted blood flow (Wilburn et al., 2021). The distortion observed in the human working muscle was postulated to be due to the inhibition of venous return during muscle contraction and represented the result of increased pressure, leading to disruption of myofiber integrity. This is also true for broilers that are characterized by rapid growth and breast muscle hypertrophy with compromised blood supply. Noticeably, heavy broilers spend much of their time lying down, which may increase pressure and breast muscle contraction. Furthermore, swollen and elongated mitochondria with indistinct cristae structure were spotted in WB-affected muscles. This observation was not surprising because hypoxia has been shown to induce mitochondrial swelling (Niquet et al., 2003) and elongation (Khacho et al., 2014), and consequently leads to apoptosis/necrosis through caspase-3 activation, which has also been previously delineated in WB-affected muscles and in hypoxic myoblasts (Greene et al., 2023).

Although warranting further functional studies, the increased level of Ca^2+^ in both WB-affected muscles and hypoxic myoblasts suggests that calcium overload is likely a leading cause for the mitochondrial dysfunction. The first supporting argument for the abovementioned hypothesis is that mitochondria can uptake Ca^2+^ through at least two routes, a uniporter (Gunter et al., 1998; Rizzuto et al., 1998; Kirichok et al., 2004) and a rapid uptake pathway (Sparagna et al., 1995), whose molecular nature and mechanisms still elude us. The second rationalization is that hypoxia has been shown to induce Ca^2+^ accumulation in mitochondria through the reversal of the Na^+^-Ca^2+^ exchanger (Haigney et al., 1992; Griffiths and Halestrap, 1993; Chacon et al., 1994; Miyamae et al., 1996; Griffiths et al., 1998; Kushwaha et al., 2023). The third assertion is that mitochondrial Ca^2+^ overloading triggers the opening of the mitochondrial permeability transition pore (Giorgio et al., 2017), a non-specific pore in the mitochondria inner membrane, allowing the entry of water and solutes into the mitochondrial matrix, and thereby resulting in mitochondrial swelling and disruption of cristae and outer membrane.

Of vested interest, Ca^2+^ and ROS are considered the main transduction signals linking the ER and mitochondria and help them to adapt their response to hypoxia in a tightly regulated manner (Yan et al., 2008). In a previous study, using primary myoblasts, we have shown that hypoxia induced ER stress, which was also evident in WB-affected muscle (Greene et al., 2023), suggesting that ER-mitochondria tethering could be dysregulated. The significant decline of MFN1 protein levels in both WB-affected muscles and hypoxic myoblasts supports the abovesaid hypothesis. In addition to its key role in mitochondrial dynamics, MFN1/2 were found to be located on both the ER and mitochondrial membranes (Chen et al., 2012; Liu and Zhu, 2017), where it interacts with other proteins and mediates ER-mitochondria tethering (Filadi et al., 2015; Kirshenbaum et al., 2024). Genetic manipulation studies demonstrated critical roles for MFNs in maintaining a physical juxtaposition of the ER and mitochondria and a normal inter-organelle Ca^2+^ signaling (Han et al., 2021; Chen et al., 2024). In addition, the upregulated expression of the calcium channels, ITPR1/2, supports the notion of increased transfer of Ca^2+^ from the ER to the mitochondria and its mitochondrial accumulation (Wiel et al., 2014). Inositol 1,4,5-trisphosphate receptor (ITPRs) are parts of contact sites between mitochondria and ER, which are known as mitochondria-ER contacts (MERCs) or mitochondria-associated ER membranes (MAMs) (Pinton, 2018). In fact, ITPRs interact with VDAC of the outer mitochondrial membrane through the molecular chaperone glucose-regulated protein, GRP75, to form a tripartite complex that is critical for mitochondria-ER coupling, crosstalk, and tethering (Lee et al., 2019; Ziegler et al., 2021).

The close contacts between the ER and mitochondria became widely appreciated as important dynamic platforms that provide an excellent scaffold for communication and crosstalk between the two organelles and play key roles in different signaling pathways. This allows rapid exchanges of biological molecules, such as Ca^2+^ transfer, to maintain cellular homeostasis and organelle function. It has been shown that ER-mitochondria contact also stimulates mitochondrial division (Friedman et al., 2011), which is required for many cellular processes, including metabolic adaptation (Mishra and Chan, 2016), and any defect in this contact may affect mitochondrial dynamics and leads to pathologies (Nunnari and Suomalainen, 2012). Here, we showed that INF2 expression was significantly upregulated in both WB-affected muscles and hypoxic myoblasts, indicating an alteration of mitochondrial division. Inverted formin 2 (INF2) polymerizes actin at the ER, enhances ER-mitochondria contact, and recruits DNM1 (which was also upregulated in WB-affected muscles), leading to a ring formation and subsequent mitochondrial outer membrane (OMM) contraction (Korobova et al., 2013; Fung et al., 2023). Mitochondrial division also requires the division of the inner mitochondrial membrane (IMM) (Chakrabarti et al., 2018). The concomitant increase of the IMM mitochondrial gatekeepers OMA1 and s-OPA1 proteins, at least in WB-affected muscles, suggested a mitochondrial fission status, which is supported by mitochondrial fragmentations observed by the EM analysis. However, the elongated mitochondria also observed in our experimental conditions by EM indicated two potential scenarios: 1) these elongated mitochondria were protected from being degraded by mitophagy (Gomes and Scorrano, 2011), or 2) they were elongated to be ready for division (Scott and Youle, 2010). Overall, it is known that when the transmembrane potential across the IMM is lost, long L-OPA1 is cleaved to short fusion-inactive s-OPA1 isoforms by the mitochondrial stress-sensitive protease OMA1, causing mitochondria fission and fragmentation (Zhang et al., 2014; Gilkerson et al., 2021). Although OPA1 protein expression did not change in hypoxic myoblasts, the upregulation of SPIRE supported mitochondrial fission status (Manor et al., 2015) and suggested differential fission pathways between *in vivo* and *in vitro* models, with the same outcome and endpoint as hypoxia has been shown to induce mitochondrial fission (Nishimura et al., 2018; Yuan et al., 2021; Gillmore et al., 2022).

It is well known that in high-energy demanding tissues, like muscle, calcium homeostasis is intrinsically associated to ATP production both via the Krebs cycle and OXPHOS to regulate cellular bioenergetics (Glancy and Balaban, 2012). It is, therefore, expected that hypoxia, mitochondrial Ca^2+^ overload, and defects in mitochondrial dynamics and tethering would affect mitochondrial biogenesis, function, and bioenergetics. The upregulation of mtDNA D-loop and PPARγ expression on one hand (in WB muscle and hypoxic myoblasts), and the downregulation of PGC-1α (in hypoxic myoblasts) on other hand, indicated that mitochondrial biogenesis was altered. PGC-1 is considered as the master regulator of mitochondrial biogenesis by virtue of its ability to control the expression of several critical transcription factors, and its inhibition has been reported to alter mitochondrial biogenesis (Gureev et al., 2019; Jamwal et al., 2021). The increased expression of mtDNA D-loop is intriguing, but it was probably associated with increased number of mitochondria due to fission and/or release of mtDNA following mitochondrial fragmentation (Bao et al., 2019).

Similarly, the dysregulation of av-UCP, ANT1, and Ski genes indicated that mitochondrial function was affected. In contrast to mammals where at least 5 UCPs have been discovered, only one av-UCP has been characterized in avian species (Raimbault et al., 2001). Although its physiological roles are still largely unknown, av-UCP, a member of the mitochondrial anion carrier family, was postulated to be involved in thermogenesis, redox balance, and ROS (Dridi et al., 2004; Davoodi et al., 2023). Among the four ANT paralogous, ANT1, located in the IMM, acts as a gated pore that allows ADP to enter the mitochondria and ATP to exit (Chen et al., 2023). Ski has been found to enhance the activity of cytochrome C oxidase and citrate synthase and stimulates mitochondrial biogenesis and fatty acid beta oxidation (Ye et al., 2011). Mammalian and rodent studies showed that UCPs (UCP2/3) are key uniport mechanisms for mitochondrial Ca^2+^ uptake (Trenker et al., 2007), as well as major promoters for mitochondrial proton leak and inefficient OXPHOS (Parker et al., 2008). Approximately two-thirds of the basal proton conductance is correlated to ANT abundance, which has been reported to stimulate mitochondrial proton leak (Bertholet et al., 2019). While direct evidence is limited, research suggested that Ski, through the TGFβ signaling pathway, might alter mitochondrial function and increase proton leak (Tecalco-Cruz et al., 2018).

Together, all the abovementioned mitochondrial abnormalities (morphology, structure, tethering, biogenesis, etc.) point to a defect in OXPHOS and ATP production. The dysregulated expression of genes and proteins associated with mitochondrial complexes I to IV support this hypothesis. Abundances of complex I components, NDUFV2 (N module, hydrophilic arm) and mtND4 (P module, hydrophobic arm) mRNA were significantly decreased in both WB-affected muscle and hypoxic myoblasts, suggesting a decreased activity of complex I, which oxidizes NADH and transfers electrons to ubiquinone in a reaction coupled with proton pumping (Gutiérrez-Fernández et al., 2020). Complex I is pivotal in maintaining metabolic homeostasis by sensing O_2_ and initiating response to mitochondrial stress, such as hypoxia (Fernández-Agüera et al., 2015). Previous studies have reported that hypoxia decreased complex I activity, which was also associated with increased mitochondrial ROS (Keeney et al., 2006; Grivennikova and Vinogradov, 2013; Papa and De Rasmo, 2013). The increased expression of the complex II component, SDHA, suggested an activation of complex II due to complex I deficiency. In that regard, it has been reported that compensatory complex II activity remodel and rescue mitochondrial respiration by shifting cellular fuel sources from NADH to FADH2 (Acín-Pérez et al., 2014). The downregulation of its subunit expression (CytB, RISP, and UQCRC2 in WB-affected muscle and UQCRC2 in hypoxic myoblasts) indicated lower activity of complex III, which is the central collector delivering electrons through cytochrome c to complex IV. It has been shown that cells with defects in complex III have decreased activity of complex I (Acín-Pérez et al., 2004). Furthermore, it has been reported in a human cellular model that a small frame deletion in CytB was associated with a severe impairment of respirasome (complexes III, I and IV) assembly and ROS production (Tropeano et al., 2020). Complex IV is the terminal complex in the ETC, transferring electrons from ferro-cytochrome c to molecular oxygen, converting the latter to water (Li et al., 2006). MT-CO1 is encoded by the mitochondrial genome and serves as the main integral membrane catalytic subunit of complex IV. Its downregulation in our experimental conditions in both WB-affected muscles and hypoxic myoblasts indicated a decreased activity of complex IV that likely leads to increased oxidative stress (Shen et al., 2018) and altered ATP production (Belevich et al., 2007). Reduced MT-CO1 was found to be associated with dysregulation of mitochondrial biogenesis and lower expression of PGC-1, which have also been seen in this study.

The mitochondrial complex V or ATP synthase is the fifth multi subunit OXPHOS complex that synthesizes ATP from ADP (Nijtmans et al., 1995). It is a dual motor that is composed of two molecular units, the cytoplasmic catalytic F1 and the membrane-embedded F0 that allows proton channeling (Forgac, 2007). Although the low basal and maximal respiration and ATP production were expected in hypoxic cells (Heerlein et al., 2005; Flood et al., 2023) and WB-affected tissue (Zhang et al., 2024), the increased ATP synthase activity in isolated mitochondria from WB-affected was puzzling. While principles and mechanisms of intracellular energy distribution as well as interaction of ATP-producing systems remain poorly understood particularly in avian species, it is probable that the observed increase in ATP synthase activity was associated with high levels of AMP/ADP and/or Ca^2+^. It is well accepted that ATP synthase can allosterically be activated by AMP, which is also known to activate AMPK and both AMP content and phosphorylated AMPK have been reported to be higher in WB-affected tissues (Zhang et al., 2020; Zhang et al., 2024). Despite the fact that no molecular mechanism has yet been described, intramitochondrial Ca^2+^ has been reported to directly activate F1-F0-ATPase (Harris, 1993; Scholz and Balaban, 1994; Territo et al., 2000). Several studies have demonstrated that Ca^2+^-dependent mitochondrial dehydrogenases (PDH, ICDH, and KDH) are potential routes for Ca^2+^-dependent ATP synthase activation (Denton et al., 1972; Kerbey et al., 1976; Denton et al., 1978; McCormack and Denton, 1979; Lawlis and Roche, 1980; Rutter and Denton, 1989; McCormack et al., 1990).

In summary, regardless of some differences in gene/protein expression (SPIRE, OMA1, OPA1, etc.) between *in vivo* (WB muscles) and *in vitro* (hypoxic cells) studies, overall, it is clear that mitochondrial morphology, biogenesis, dynamics, tethering, function, and bioenergetics were all affected. Our study is the first, to the best of our knowledge, providing fundamental insights related to mitochondrial dysfunction in relatively new muscle myopathy with unknown etiology, and suggesting that Ca^2+^ signaling might play a key role that needs further in-depth investigations. It is also worth noting that several mRNA abundances and protein levels, such as OMA1 and MTCO1, were not perfectly correlated, which is not surprising, and this might due to RNA decay and stability, protein half-life and stability, translation efficiency, and/or likely other complex regulatory pathways (post-translational modifications) that can decrease protein production even when the mRNA levels are high (Maier et al., 2009).

## Data Availability

The data that support the findings of this study are available from the corresponding author upon reasonable request.
